# Nasal Chondromesenchymal Hamartoma (NCMH): a systematic review of the literature with a new case report

**DOI:** 10.1186/s40463-015-0077-3

**Published:** 2015-07-03

**Authors:** Katrina Anna Mason, Annakan Navaratnam, Evgenia Theodorakopoulou, Perumal Gounder Chokkalingam

**Affiliations:** Barts and The London School of Medicine and Dentistry, The Blizard Institute of Cell and Molecular Science, 4 Newark Street, Whitechapel, E1 2AT London, UK; Colchester Hospital University NHS Foundation Trust, Colchester, UK

**Keywords:** Nasal neoplasms, Hamartoma, DICER1 protein human, Review

## Abstract

**Background:**

Nasal chondromesenchymal hamartoma (NCMH) is a very rare, benign tumour of the sinonasal tract usually presenting in infants. We present a systematic review of NCMH cases alongside a case report of an adult with asymptomatic NCMH.

**Methods:**

A systematic review was conducted in accordance with PRISMA guidelines. A PubMed, EMBASE and manual search through references of relevant publications was used to identify all published case-reports of NCMH. Data was collected from each case-report on: patient demographics, laterality, size and location of NCMH, presentation, co-morbidities, investigations, treatment and follow-up.

**Results:**

The systematic review identified 48 patients (including ours): 33 male, 15 female. Mean age was 9.6 years (range: 1 day–69 years) with the majority aged 1 year or younger at presentation (*n* = 18). Presentations included: nasal congestion (*n* = 17), nasal mass (*n* = 15) and eye signs (*n* = 12). NCMH also involved the paranasal sinuses (*n* = 26), orbit (*n* = 16) and skull-base (*n* = 14). All patients underwent operative resection of NCMH. A small 2014 case-series found DICER1 mutations in 6 NCMH patients, establishing a link to the DICER1 tumour spectrum.

**Conclusions:**

NCMH is a rare cause of nasal masses in young children and adults. In light of the newly established link between NCMH and DICER1 mutations surgeons should be vigilant for associated DICER1 tumours, as NCMH may be the ‘herald tumour’ of this disease spectrum.

## Background

Nasal chondromesenchymal hamartoma (NCMH) is a very rare, benign tumour of the sinonasal tract. Forty-seven cases have been reported in the English literature and the vast majority of these presentations are in infants and young children often below the age of one. NCMHs have a mixed morphological structure comprised of predominantly mesenchymal and cartilaginous components. NCMH patients present with symptoms that are dependent on the location of the tumour in the nasal cavity or paranasal sinuses and their compression of local structures. These symptoms range from nasal obstruction to visual impairment and facial and dental pain. To date there have only been 6 cases of adult presentation of NCMH. Here we present the first systematic review of NCMH cases published in the literature to assess the patient demographics, presentation, management and prognosis of NCMH alongside a new and unusual case in an adult.

### Case report

A 49-year-old man was referred to our outpatient clinic presenting with a small mass in his right nasal cavity. The lump, which the patient first noticed 5 years ago, had been growing insidiously over time and by the time of presentation had become visible at the right anterior naris. The patient did not complain of any symptoms but sought consultation as his wife was concerned by the cosmetic appearance of the mass.

Examination revealed a large firm mass arising from the right side of the anterior nasal septum with approximately 0.5 cm attachment to the anterior cartilage of the septum. The left and right nasal cavities were otherwise unremarkable. Clinically, the mass had the appearance of a papilloma confined to the nasal cavity with an attachment to the septum only and therefore further imaging was not undertaken. The differential diagnoses considered at the time of presentation were: nasal polyp, squamous papilloma or inverted papilloma.

**Fig. 1:**
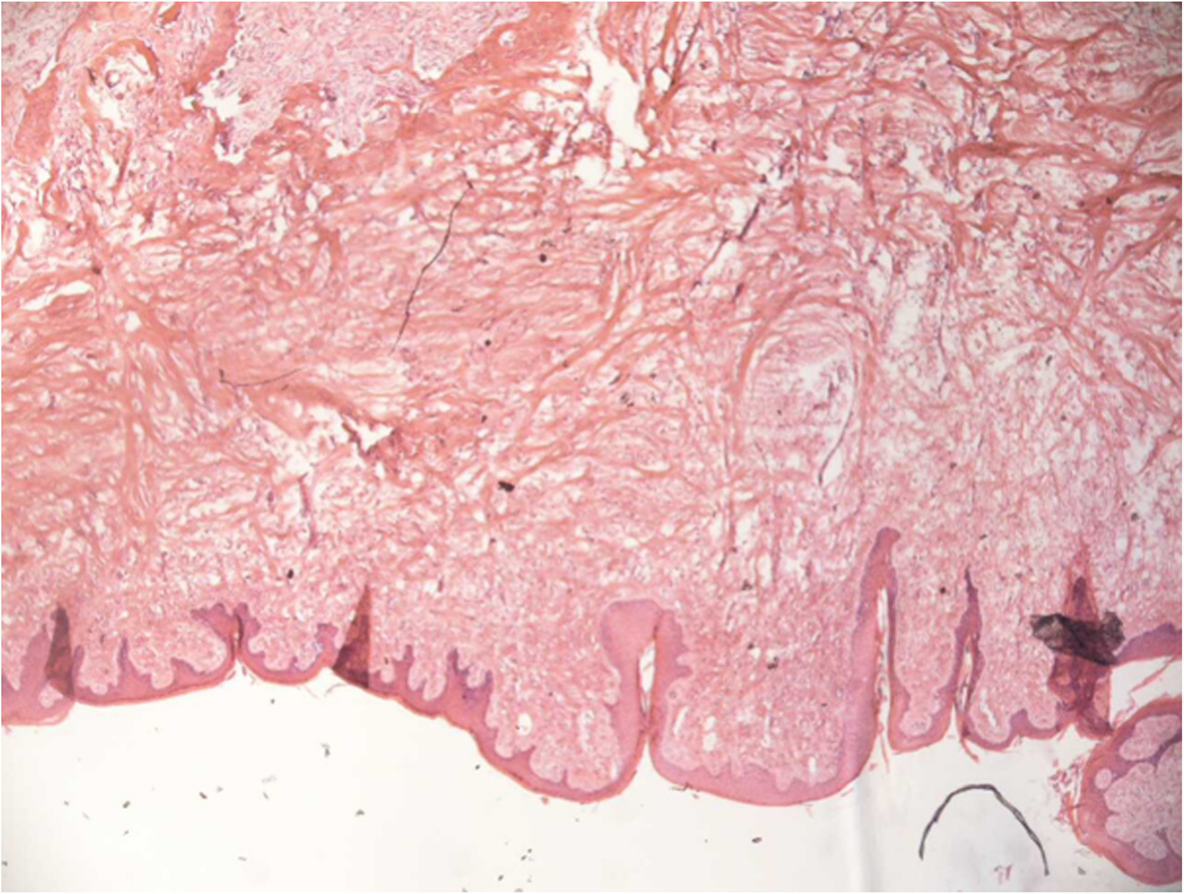
Nodule containing cartilage and aneurysmal bone and covered by stratified squamous epithelium with keratinisation (magnification ×25, haematoxylin & eosin stain)

**Fig. 2:**
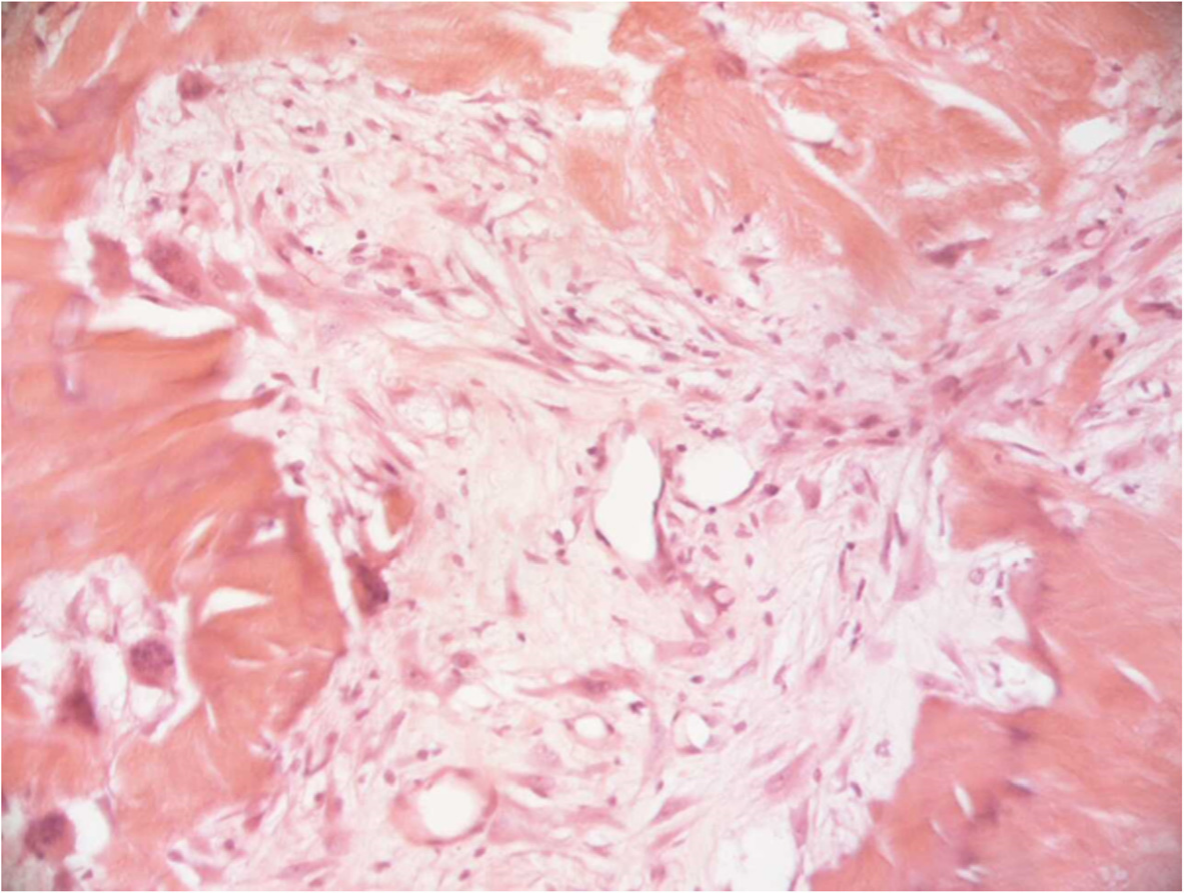
Areas of osteoid formation (magnification ×200, haematoxylin & eosin stain)

The patient subsequently underwent excisional biopsy of the right nostril mass under general anaesthetic using a circumferential subperichondrial incision with a small margin. Intraoperatively, the mass had the macroscopic appearance of a 0.5 cm × 2 cm × 2 cm calcified nodule. Due to a small 0.5 cm base, subsequent healing was achieved by secondary intention aided by the routine application of topical antibacterial cream. Histopathological analysis showed the nodule to contain cartilage and aneurysmal bone covered in stratified squamous epithelium with keratinisation (Figs. 1 and 2). Histopathological diagnosis was made using a haematoxylin & eosin stain. These findings were consistent with a diagnosis of a nasal chondromesenchymal hamartoma.

The patient was followed up in clinic and was discharged after 2 years having shown no signs of recurrence. Furthermore, a telephone interview was conducted 4 years post operation and the patient reported no recurrence of the nasal mass. He confirmed that he had no post-operative complications and was happy with the outcome of the operation.

## Methods

**Fig. 3:**
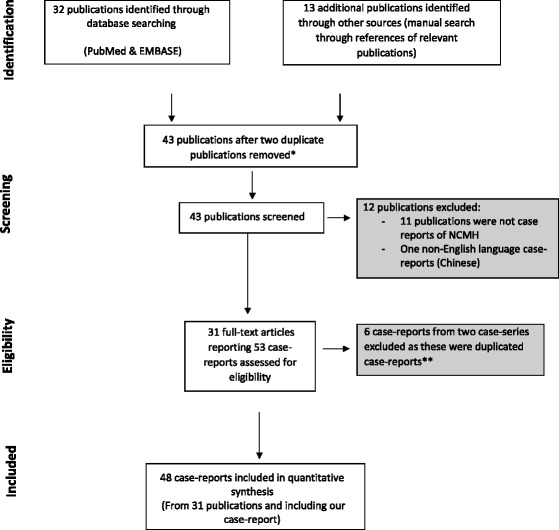
Four-phase flow diagram of systematic review in accordance with PRISMA guidelines * Two individual publications of single case-reports were excluded from analysis as these had previously been published, Schultz et al. [2] and Kang et al. [3].** See Table 1

A systematic review was undertaken in accordance with PRISMA guidelines [1]. No systematic review protocol was used, however our systematic review methodology is described below and a four-phase flow diagram is represented in Fig. 3. All published case-reports of NCMH were included in the review. A PubMed search (MEDLINE) (1975 to May Week 2, 2015) was carried out using the following terms [(chondromesenchymal hamartoma) AND (nasal OR sinus OR maxillary OR ethmoid OR sphenoid OR frontal OR orbit OR cranial)]. An EMBASE search (1975 to May Week 2, 2015) was carried out using a best sensitivity-combination strategy. The PubMed search resulted in 32 citations of which 24 were relevant, 6 were not NCMH case-reports, one was a Chinese language case-report, and one case was a duplicate case-report publication [2]. An EMBASE search and a manual search through references of relevant publications yielded 11 further relevant citations. Of these only 6 were included in the analysis; one case was found to have been a duplicate case report [3, 4] and four other possible cases of NCMH were found through publication citation search, but were labelled as “Mesenchymal chondrosarcoma” [5] “nasal hamartoma” [6], “nasopharyngeal hamartoma” [7], and “congenital mesenchymoma” [8], and were therefore not included. Thirty-one publications that report 47 cases of NCMH were included in this systematic review. Data was collected on patient demographics (age, gender), laterality, size and site of NCMH, presentation, co-morbidities, investigations, treatment and follow-up. These were also the principle summary measures. Two authors performed the database search, the manual search through references of relevant publications, and extracted the relevant data from the case-reports. Data was entered into an Excel 2013 Microsoft Office™ database which was used to carry out basic statistical analysis.

### Results of systematic review

Forty-Eight NCMH patients (including our case) have been reported in the English literature (Fig. 3). Most cases presented in males; 33 male and 15 female, with a male to female ratio of 2.2:1 ratio. The mean age was 9.6 years (range: 1 day–69 years). A large proportion of these patients were aged 1 year or younger at presentation (*n* = 18) and only 8 adult patients (including our case) have been described. Site of pathology was limited to the nasal cavity only in 10 patients, and involved the paranasal sinuses (maxillary, ethmoid, sphenoid) in 26 patients, the orbit in 16 patients, extending to the skull base in 14 patients, had intracranial extension in 8 cases, involved the nasopharynx in 3 patients and the oropharynx in 2 patients (see Table 1).

**Table 1:** Summary table of systematic review of NCMH cases reported in the literature

Author, Publication date	Case No	Age D/M/Y	Sex	Side & Size	Site	Symptoms	Co-morbidity	Investigations	Treatment	Follow Up/
(1) McDermot, 1998 [10] USA	1	5 D	M	ND	1. Nasal cavity	1. Nasal Mass	ND	CT	Surgical excision	No recurrence at2 years
						2. Respiratory Difficulties				
	2	3 M	F	ND	1. Nasal cavity	1. Nasal Mass	ND	MRI	Surgical excision	No recurrence at 2 years
					2. Ethmoid Sinus	2. Otitis Media				
					3. Intracranial extension					
	3	3 M	M	ND	1. Nasal cavity	1. Choanal Mass	ND	ND	Biopsy then surgical excision Subsequent chemotherapy	No recurrence at 4 years
						2. Respiratory distress				
	4	2 M	M	ND	1. Nasal cavity	1. Nasal Mass	ND	ND	Surgical excision	No recurrence at 18 months
					2. Intracranial extension					
	5	12 D	F	ND	1. Nasal cavity	1. Nasal Mass	ND	CT	Surgical excision & further re-excision after 16 months	Unchanged persistent tumour in superior nasal cavity at 12 months
					2. Intracranial extension					
	6	14 D	M	ND	1. Nasal cavity	1. Nasal Mass	ND	CT	Surgical excision VP shunt for hydrocephalus	Residual tumour in anterior cranial fossa at 9 months
					2. Ethmoid sinus	2. Hydrocephalus & agenesis of corpus callosum				
					3. Intracranial extension					
	7	7 Y	M	ND	1. Nasal cavity	1. Nasal Mass	PPB	ND	Surgical excision	No recurrence at 2 months NB later reported by Priest et al. 2010 [27]- showed with multiple recurrences in first 3 years
					2. Sphenoid sinus	2. Nasal Congestion				
(2) Chae 1999 Korea [30] *abstract only, Korean paper	8	3 M	F	Right Size: 3.5 × 7.5 × 2.5 cm	1. Nasal cavity	1. Epistaxis	ND	CT	Surgical excision	ND
					2. Ethmoid sinus	2. Obstruction				
					3. Cribriform plate					
(3) Kim D 1999 [31] USA	9	3 M	F	Right Size: ND	1. Nasal cavity	1. Nasal mass	None stated	CT MRI	Surgical excision with mid-facial de-gloving and bi-frontal craniotomy	No recurrence at 18 months
					2. Intracranial extension	2. Otitis media				
					3. Ethmoid sinus					
(4) Kato, 1999 [17] Japan	10	4 M	M	Left Size: ND	1. Nasal cavity	1. Nasal Mass	None stated	CT	Two stage surgical excision 1st intracranial/sinus lesion, 2nd intranasal lesion	No recurrence at 13 years
					2. Intracranial extension	2. Respiratory distress with cyanosis when feeding				
					3. Extension to left orbit	3. Opthalmoplegia left eye			Radiotherapy post op	
(5) Hsueh 2001 [32] Taiwan (2 cases)	11	0 D	M	Left Size: ND	1. Nasal cavity	1. Left facial swelling	None stated	CT MRI	Excision biopsy then subsequent surgical excision with lateral rhinotomy and craniofacial approach	Recurrence after excision biopsy No recurrence at 5 years after second surgery
					1. Sphenoid sinus	2. Left nasal mass				
					3. Ethmoid sinus	3. Respiratory distress & cyanosis when feeding				
					4. Compression of left orbit					
						4. Proptosis on recurrence				
	12	9 M	M	Right ND	1. Nasal cavity	1. Asymmetric face	None stated	CT MRI	Surgical resection	No recurrence at 9 months
					2. Maxillary sinus	2. Right opthalmoplegia, enopthalmos and hypotropia				
(6) Alrawi 2003 [14] Ireland	13	16 Y	M	Left 1.5 × 1.5 cm	1. Nasal cavity	1. Nasal swelling	None stated	CT MRI	Surgical resection with delayed reconstruction with forehead flap	No recurrence at 8 months
(7) Shet, 2004 [21] India	14	1 Y	M	Left Size: ND	1. Nasal cavity	1. Proptosis of left eye	Non stated	CT	Chemotherapy (VID) as biopsy suggested spindle cell sarcoma- 30 % reduction in tumour size Then Left maxillectomy and surgical excision	Residual tumour at 1.5 years near eye but no further re-growth & stable
					2. Extension into left orbit	2. Left facial swelling				
					3. Ethmoid sinus					
					4. Sphenoid sinus					
(8) Kim B, 2004 [22] Korea	15	5 M	M	Left Size: ND	1. Nasal cavity	1. Left eye ptosis	None stated	CT	Frontal craniotomy & trans-nasal surgical resection	ND
					2. Compression of left orbit					
					3. Defect left ethmoidal bone					
					4. Defects anterior cranial fossa					
(9) Norman, 2004 [15] USA	16	11 Y	M	Left Size: ND	1. Nasal cavity	1. Headaches left sided	None stated	CT	Endoscopic biopsy and anterior craniofacial resection	ND
					2. Displacement left orbital wall					
(10) Ozolek, 2005 [11] USA (4 cases)	17	11 Y	M	Left Size: ND	1. Nasal cavity	1. Nasal mass	None stated	ND	Surgery and care undertaken in another hospital	ND Surgery and care undertaken in another hospital
					2. Ethmoid sinus					
					3. Extension into left orbit					
	18	17 Y	F	ND Size: ND	1. Nasal cavity	1. Nasal obstruction	None stated	ND	Surgical excision	ND
						2. Facial pain				
	19	25 Y	M	Bilateral 8 × 5 × 3.5 cm	1. Nasal cavity	1. Respiratory distress from obstructing oropharyngeal tumour requiring emergency tracheostomy	1. Multiple Intracranial vascular aneurysms 2. Longstanding nasopharyngeal tumour- biopsy aged 13 ‘chronic inflamed polyp’	CT	Multiple surgical resections within one year including, tracheostomy and initial surgical resection, further surgical resection of bulbar mass and nasal tumour, then Le-Fort osteotomy and further surgical resection	ND
					2. Maxillary sinus					
					3. Nasopharynx					
					2. Oropharynx	2. Chronic sinusitis				
	20	69 Y	F	Right Size: ND	1. Nasal cavity	ND	None stated	ND	Surgical excision	ND
					2. Ethmoid sinus					
(11) Low 2006 [33] UK	21	11 Y	M	Right Size: ND	1. Nasal cavity	1. Nasal Obstruction	None stated	CT	Surgical excision	No recurrence at 2 months
						2. Epistaxis				
(12) Johnson, 2007 [29] USA	22	15 Y	F	Bilateral Size: ND	1. Nasal cavity	1. Nasal obstruction	1. PPB	CT	Endoscopic surgical excision	No recurrence at 6 months
					2. Nasopharynx	2. Chronic sinusitis	2. Sertoli-Leydig cell Ovarian Tumour			
							3. Congenital phthisi bulbi			
(13) Silkiss, 2007 [18] USA	23	7 M	M	Right 3.2 × 1.4 cm	1. Nasal cavity	1. Ptosis	None stated	CT MRI	Surgical resection- right lateral rhinotomy	No recurrence at 18 months
					2. Erosion of cribriform plate	2. Extropia				
					3. Compression of right orbit	3. Strabismus 4. Stertor				
(14) Nakagawa 2008 [34] Japan	24	12 Y	M	Left 1.5 cm	1. Nasal cavity	1. Nasal obstruction	None stated	CT	Endoscopic surgical resection and further endoscopic surgical resection after recurrence	Recurrence at 2 months No recurrence at 5 months post second surgery
					2. Sphenoid sinus					
					3. Ethmoid sinus					
					4. Maxillary sinus					
(15) Finitsis, 2009 [35] Greece	25	12 M	M	Left 4 cm × 4.2 cm	1. Nasal cavity	1. Respiratory distress	None stated	CT MRI	Pre-operative embolization Then Surgical resection with midface de-gloving	ND
					2. Compression of left orbit					
					3. Maxillary sinus compression					
					4. Nasopharynx					
(16) Kim J, 2009 [23] Korea	26	19 M	M	Left 2.7 × 3.5 cm	1. Nasal cavity	1. Watery rhinorrhoea	None stated	CT MRI	Endoscopic surgical resection ×2	Recurrence at one year; 2nd surgery. No recurrence 10 months after second surgery
					2. Orbital extension	2. Nasal Obstruction				
					3. Intracranial extension					
(17) Priest, 2010 [27] USA *case previously reported by McDermot et al. 1998 **case previously reported by Johnson et al. 2007 (2 new cases)	-	7 Y *	M	Initially unilateral, then bilateral	1. Sphenoid sinus	1. Nasal Congestion	1. PPB type II–III		Four resections over 3 years	Followed up for 13 years with multiple recurrences in first 3 years
					2. Left Nasal cavity	2. Nasal mass	2. Lung cysts			
	-	15 Y **	F	Bilateral	1. Bilateral nasal cavities	1. Chronic Sinusitis	1. PPB Type II		Surgical resection	No recurrence at 51 months
					2. Bony erosion of posterior septum	2. Facial Pain	2. Sertoli-Leydig Cell Ovarian Tumour			
					2. Extending into nasopharynx	3. Nasal Congestion	3. Congenital phthisi bulbi Stickler syndrome			
						4. Nasal obstruction				
	27	10 Y	F	Bilateral Size: ND	1. Bilateral nasal cavities	1. Nasal obstruction	1. PPB Type III	CT	Surgical resection	No recurrence at 21 months
	28	11 Y	M	Right Size: ND	1. Nasal cavity	1. Nasal obstruction	1. PPB Type III	ND	Surgical resection	No recurrence at 4 months
					2. Extension to anterior skull base					
(18) Sarin, 2010 [24] India	29	2.5 Y	M	Right Size: ND	1. Nasal cavity	1. Right eye oculomotor impairment	ND	MRI	Biopsy and then lateral rhinotomy for excision	ND
					2. Maxillary, ethmoid and sphenoid sinus					
					3. Erosion of middle wall of orbit					
(19) Eloy 2011 [25] Belgium	30	18 M*	M	Right 0.5 × 0.4 cm	1. Nasal cavity	1. Nasal obstruction	None stated	CT MRI	Endoscopic surgical resection	ND
					2. Ethmoid sinus	2. Nasal mass				
					3. Extension into right orbit	3. Hypertelorism, proptosis, diplopia				
					4. Intracranial extension	4. Nasal swelling *symptoms 1st noticed at 2 months- delayed referral from Algeria to Brussels				
(20) Jeyakumar 2011 [26] USA	31	7 D	F	Right Size: ND	1. Nasal cavity	1. Nasal Mass	None stated	CT MRI	Endoscopic surgical excision	ND
						2. Stertor				
						3. Proptosis right eye				
(21) Mattos 2011 [20] USA	32	3 Y	M	Left Size : ND	1. Nasal cavity	1. Eye infections	None stated	CT MRI	Endoscopic excision the further surgical excision	Recurrence at 21 months required further resection
					2. Ethmoid sinus	2. Eye congestion				
					3. Extension into right orbit	3. Nasal obstruction				
					4. Left maxilla	4. Cheek fullness				
						5. Intermitted left eye/face pain				
(22) Behery, 2012 [36] USA	33	11 Y	M	ND	ND	Nasal Obstruction	1. PPB		Surgical resection	ND
(23) Uzomefuna, 2012 [37] Ireland	34	8 Y	M	ND	1. Sphenoid sinus	1. Frontal Headache	ND	CT MRI	Endoscopic surgical resection	No recurrence at 6 months
					2. Ethmoid sinus					
(24) Cho, 2013 [4] Korea	35	14 Y	M	Left 5 cm × 5.3 cm × 4 cm	1. Nasal cavity	1. Swelling and pain to left face	None stated	CT	Subtotal maxillectomy, removal or orbital floor, removal of medial nasal mucous membrane. Reconstruction with iliac crest bone block	No recurrence at 4 years
					2. Maxillary sinus	2. Tooth mobility				
					3. Intraoral					
					4. Orbital floor destruction					
(25) Li Y, 2013 [12] China	36	40 Y	F	Bilateral Size: ND	1. Nasal Cavity	1. Nasal Obstruction	None stated	CT MRI	Complete radical resection	Recurrence at 3 months *Malignant transformation seen on histology
					2. Maxillary sinus	2. Bloody rhinorrhoea				
					3. Ethmoid sinus					
(26) Li GY 2013 [9] China	37	23 Y	M	Left 3.2 × 2.5 cm	1. Naval cavity	1. Left Lacrimal Sac	None stated	ND	Endoscopic surgical excision	No recurrence at 3 months follow up
					2. Extension to lacrimal sac & left orbit	2. Proptosis				
					3. Ethmoid sinus	3. Lateral displacement of globe				
(27) Moon, 2014 [19] Korea	38	9 M	F	Right Size: ND	1. Nasal cavity	1. Incomitant esotropia of right eye (inability to abduct right eye) No nasal symptoms	None stated	CT MRI	Surgery and care undertaken in another hospital	ND Surgery and care undertaken in another hospital
					2. Maxillary sinus					
					3. Erosion of orbital wall					
					4. Erosion of cribriform plate					
(28) Wang T, 2014 [38] China (2 cases)	39	5 Y	M	Right 2.5 × 3.6 × 4.3 cm	1. Nasal cavity	1. Recurrent sinusitis	None stated	CT MRI	Surgical resection	No recurrence at 3 years
					2. Ethmoid sinus	2. Nasal Obstruction				
					3. Intracranial extension					
	40	6 W	F	Left 2.6 × 3.4 × 3.9 cm	1. Nasal cavity	1. Nasal obstruction	None stated	CT MRI	Surgical resection	No recurrence at 10 months
					2. Pressure remodelling of adjacent bones	2. Watery rhinorrhoea				
(29) Obidan, 2014 [39] Saudi Arabia	41	14 Y Size: ND	M	Bilateral	1. Bilateral nasal cavities	1. Nasal Obstruction	1. PPB	CT	Surgical endoscopic resection	ND
						2. Decreased sense of smell				
(30) Stewart, 2014 [13] USA **4 patients previously reported by Priest et al. 2010 [27] (4 new cases)	-	7 Y**	M	Initially unilateral, then bilateral	ND	1. Nasal Congestion	1. PPB		Surgical resection	Multiple recurrences
							2. Lung cysts			
	-	15 Y**	F	Bilateral	ND	1. Chronic Sinusitis	1. PPB		Surgical resection	No recurrence
						2. Facial Pain	2. Sertoli-Leydig Cell Ovarian Tumour			
						3. Nasal Congestion				
	-	10 Y**	F	Bilateral	ND	1. Nasal congestion	1. PPB		Surgical resection	No recurrence
	-	11 Y**	M	Right	ND	1. Nasal Congestion	1. PPB		Surgical resection	No recurrence
	42	8 Y	M	ND Size: ND	ND	1. ND	1. PPB	ND	Surgical resection	No recurrence
							2. Pulmonary cysts in utero			
							3. Jejunal Polyps			
	43	13 Y	F	Bilateral Size: ND	ND	1. ND	1. PPB	ND	Surgical resection	No recurrence
							2. Thyroid Papillary carcinoma			
							3. Sertoli-Leydig tumour			
	44	8 Y	M	Bilateral Size: ND	ND	1. Chronic Sinusitis	1. PPB	ND	Surgical resection	No recurrence
	45	6 Y	F	ND Size: ND	ND	ND	1. PPB	ND	Surgical resection	No recurrence
							2. Left cystic nephroma			
							3. Small bowel loop			
	46	21 Y	F	Right Size: ND	ND	1. Nasal Congestion	1. PPB	ND	Surgical resection	Recurrence at 4 years
						2. Septal deviation	2. Sertoli-Leydig Tumour			
						3. Nasal Obstruction (at recurrence)	3. Multi-nodular goitre			
(31) Chandra 2014 [40] India	47	12 Y	M	Right Size: ND	1. Nasal cavity	1. Nasal Obstruction	None stated	CT MRI	Surgical excision	No recurrence at 5 months
					2. Ethmoid sinus	2. Proptosis				
					3. Extension into right orbit	3. Right facial pain				
(32) Mason 2015 UK	48	49 Y	M	Right 0.5 × 2 × 2 cm	1. Nasal cavity	1. Nasal mass	None stated	None	Surgical excision	No recurrence at 4 years

*Y* Years, *M* Months, *D* Days, *M* Male, *F* Female, *ND* not documented, *PPB* Pleuropulmonary blastoma, *CT* Computed Tomography, *MRI* Magnetic Resonance Imaging

Clinical presentations of NCMH patients included: nasal congestion or obstruction (*n* = 17), nasal mass (*n* = 15), eye signs (proptosis, hypotropia, enopthalmos, strabismus, exotropia etc.) (*n* = 12), facial swelling (*n* = 8), headaches or facial pain (*n* = 6), stertor or respiratory distress (*n* = 8), ophthalmoplegia (*n* = 4), recurrent sinusitis (*n* = 4), rhinorrhoea (*n* = 3), otitis media (*n* = 2), epistaxis (*n* = 2), toothache (*n* = 1), hyposmia (*n* = 1), hydrocephalus (*n* = 1) and 4 patients were asymptomatic or had no signs and symptoms documented (see Table 1).

All patients underwent operative resection of NCMH and the surgical approach was dependent on disease location. One patient had pre-operative chemotherapy due to initial misdiagnosis on biopsy as spindle cell sarcoma. One patient had pre-operative embolization to reduce operative blood loss. Follow up times were included for 24 patients, mean time for follow-up was 24 months, (range 2–156 months). Eleven patients were found to have persistent disease or disease recurrence on follow up, seven required further surgery, three patients were described as stable, and no further information was given on the other patient. Li et al. described the first and only reported case of malignant transformation of NCMH [9].

Thirty-six patients had no documented past medical problems. One adult patient had a history of multiple vascular aneurysms. Eleven of the patients had been diagnosed with pleuropulmonary blastoma (PPB) prior to NCMH detection. Of these 11 patients, 5 had other co-morbidities including three with Sertoli-Lleydig cell ovarian tumours, two with pulmonary cysts, one jejunal polyps, one papillary thyroid carcinoma, one cystic nephroma and one multinodular goitre.

A potential weakness of this systematic review is the possibility of reporting bias through publication bias both within individual case reports and across the review. This reporting bias is three-fold: firstly in the incomplete publication of all the clinical aspects of the case-reports by the original authors, for example not reporting follow-up times or co-morbidities etc. Secondly it is possible that there have been cases of NCMH that have not been reported in the literature and can therefore not be included in the systematic review. Thirdly the exclusion of case-reports of “Mesenchymal chondrosarcoma” [5] “nasal hamartoma” [6], “nasopharyngeal hamartoma” [7], and “congenital mesenchymoma” [8] alongside others, which are published prior to the first description of NCMH by McDermot et al. in 1998, may have resulted in an underreporting of true NCMH cases. However without being able to retrospectively assess and re-classify the histology of these cases we feel it is appropriate to have excluded them from our analysis and conclusions. These sources of reporting biases could potentially reduce the validity of conclusions drawn in terms of not fully representing or capturing all possible cases of NCMH.

## Discussion

NCMHs are predominantly benign lesions that are locally destructive and because of their aggressive appearance can be mistaken for a malignant tumour. However NCMHs can be slow growing and therefore have a delayed presentation. Histopathologically these lesions are analogous to other mesenchymal hamartomas, and consist of islands of chondroid tissue such as hyaline cartilage, areas of calcification, and mesenchymal cellular elements such as spindle cells and myxoid stroma.

McDermott et al. were the first to recognise NCMH as a distinct clinic-pathological entity in 1998 when they described a case series of seven patients with a tumefactive process of the nasal passages and contiguous paranasal sinuses with a detectable mass in the nose [10]. In this case series, six of the seven patients were infants under the age of 3 months. As our systematic review demonstrates NCMH predominantly presents in young children and infants under the age of one, but there have now been seven case reports, including ours, of adults with NCMH up to the age of 69 [9, 11–14]. In 2013 the first and only reported case of malignant transformation of an NCMH was described in the literature [9].

In our case, the NCMH was initially thought to be a papilloma confined to the nasal septal wall and therefore further imaging was not undertaken prior to resection. However pre-operative imaging of these lesions provides valuable information regarding involvement of adjacent structures such as the paranasal sinuses, orbit and intracranial cavity. On computed tomography (CT) imaging, NCMH are typically seen as non-encapsulated, poorly defined masses often with cystic components [15]. Magnetic resonance imaging (MRI) of NCMH demonstrates a heterogeneous mass on T1 weighted images and T2 weighted images show the presence of cystic components. MRI also has the advantage of superior tissue characterisation and delineation of invasion of adjacent structures in comparison to CT [16]. Due to rarity of NCMH, even after thorough clinical and radiographic examination NCMH can be misdiagnosed, and differential diagnoses include: inverted papilloma, aneurysmal bone cysts or ossifying fibromas, nasoethmoidal encephalocoele, chondrosarcoma, nasal lymphoma, nasal glioma and rhabdomyosarcoma. Histopathological analysis following surgical resection is therefore needed for accurate diagnosis.

Patients with NCMH most commonly present with symptoms of nasal obstruction, nasal mass, or eye signs, which reflects the involvement of NCMH in the nasal passages and orbit. Ophthalmic signs include signs of globe displacement such as strabismus, extropia, hypertelorism, proptosis, enophthalmus and ophthlmoplegia, direct results of the intra-ocular extension of NCMH or ocular compression by NCMH [17–26]. There has also been a report of a patient presenting with intra-oral symptoms due to involvement of the oral cavity [4]. Patients can therefore present to otolaryngology, ophthalmology or maxillo-facial departments and doctors in these specialties should be aware of this rare pathology. In our case, the patient did not complain of any cranial, ophthalmic or nasal symptoms but was aware of a slowly enlarging nasal mass. This is most likely due to the relatively small size of the tumour at the anterior nasal septum which did not obstruct the nasal passage.

The aetiology of NCMH is thought to be due to an underlying genetic predisposition therefore accounting for the early presentation in the majority of cases. Priest et al. and Stewart et al. investigated patients with both NCMH and the rare paediatric dysembryonic sarcoma of the lung and pleura: pleuropulmonary blastoma (PPB) [13, 27]. In patients with NCMH and PPB Stewart et al. found germline *DICER1* mutations in 6 out of 8 evaluated patients, and somatic *DICER1* mutations in 2 out of those 6 patients with germline mutations [13]. This recent finding has established genetic proof of NCMH tumour association with *DICER1* mutations and Stewart et al. therefore feel that NCMH should be considered part of the *DICER1* tumour spectrum. The *DICER1* familial tumour susceptibility syndrome confers an increased risk most commonly for pleuropulmonary blastoma (PPB) but also ovarian sex cord-stromal tumours; Sertoli-Leydig cell tumor [SLCT], juvenile granulosa cell tumour [JGCT] and gynandroblastomas. Less commonly the *DICER1* tumour spectrum includes: cystic nephroma (CN), and thyroid gland neoplasia, multinodular goitres [MNG], adenomas, or differentiated thyroid cancers. The rarest observed tumours in this spectrum, alongside NCMHs, are ciliary body medulloepithelioma (CBME), botryoid-type embryonal rhabdomyosarcoma (ERMS) of the cervix or other sites, renal sarcomas, pituitary blastomas, and pineoblastomas [28]. Eleven patients in our systematic review had previous PPB and five of these also had other *DIECR1* tumours. Surgeons and physicians should therefore be aware of these disease associations and should be vigilant of a diagnosis of NCMH in patients presenting with sino-nasal or orbital symptoms who have a history of any of these tumours. Johnson et al. importantly also point out that due to its location, NCMH is more likely to present early in life than the other *DICER1* tumours [29]. Surgeons and physicians should therefore either offer *DICER1* mutation analysis if available, or ensure long-term follow up of these patients and be vigilant for associated tumours, as NCMH may be the ‘herald tumour’ of this disease spectrum.

There are also cases in the literature of children, adolescents and adults with NCMH who have had an asymptomatic infancy [9, 11, 12, 14]. This may imply that there are non-genetic components to NCMH pathogenesis. Alternatively it may simply reflect the insidious growth of the tumour or that some NCMH patients may only exhibit the phenotype later in life. However as this is an extremely rare pathology with only very recent formal association with the *DICER1* mutation, the majority of the 42 reported cases have not had formal *DICER1* mutation analysis. Therefore an association or lack thereof in the non-tested cases cannot be inferred.

Successful management of NCMH entails complete resection in order to prevent recurrence. A complete excision however is not always technically feasible, especially in cases of intracranial extension of NCMH. An incomplete resection poses the risk of recurrence as well as the possibility of continued tumour growth and progressive symptoms. Nine patients in this systematic review were found to have disease recurrence, most likely from incomplete surgical excision.

## Conclusions

We present an unusual case of NCMH in an adult without nasal obstructive symptoms due to the anatomical location of the NCMH attached to the nasal septum. A systematic review of the literature has highlighted that presentation is mostly related to tumour location, with nasal mass, nasal obstruction and ophthalmic signs being the most common forms of presentation. The majority of patients presenting with NCMH are children and infants below the age of one, but there have now been a few adult cases of presentation. Surgical resection is the treatment of choice with low recurrence rates in the majority of cases. There has only been one reported case of malignant transformation and NCMH is still considered a benign tumour. NCMH’s association with the *DIECR1* mutation has very recently been established and therefore in light of this any patient with a *DICER1*-related tumour spectrum and new nasal or orbital symptoms should raise the suspicion of NCMH. Furthermore surgeons should subsequently be vigilant for associated *DICER1* related tumours, as due to their location NCMHs may be the ‘herald tumour’ for this disease spectrum. This case and systematic review highlights the fact that NCMH can mimic other benign and malignant lesions and that surgeons and physicians should be aware of rare pathologies accounting for nasal masses.

## Consent

Written consent was obtained from the patient for publication of this case report and accompanying images. A copy of the written consent is available for review by the editor-in-chief of this journal.

## Abbreviations

NCMH: Nasal chondromesenchymal hamartoma
EMBASE: Excerpta medica database
CT: Computed tomography
MRI: Magnetic resonance imaging
PPB: Pleuropulmonary blastoma
SLCT: Sertoli-leydig cell tumour
JGCT: Juvenile granulosa cell tumour
CN: Cystic nephroma
MNG: Multinodular goitres
CBME: Ciliary body medulloepithelioma
ERMS: Embryonal rhabdomyosarcoma
DICER1: is not an abbreviation, but the name of gene located on chromosome 14 at position q32.13
